# Truke, a web tool to check for and handle excel misidentified gene symbols

**DOI:** 10.1186/s12864-017-3631-8

**Published:** 2017-03-21

**Authors:** Izaskun Mallona, Miguel A. Peinado

**Affiliations:** Health Research Institute Germans Trias i Pujol (IGTP), Program for Predictive and Personalized Medicine of Cancer, Can Ruti Campus. Ctra. de Can Ruti, camí de les escoles, s/n, Badalona, 08916 Spain

**Keywords:** Gene symbol, Excel, Structured data, Data conversion, Machine readability

## Abstract

**Background:**

Genomic datasets accompanying scientific publications show a surprisingly high rate of gene name corruption. This error is generated when files and tables are imported into Microsoft Excel and certain gene symbols are automatically converted into dates.

**Results:**

We have developed Truke, a fexible Web tool to detect, tag and fix, if possible, such misconversions. Aside, Truke is language and regional locale-aware, providing file format customization (decimal symbol, field sepator, etc.) following user’s preferences.

**Conclusions:**

Truke is a data format conversion tool with a unique corrupted gene symbol detection utility. Truke is freely available without registration at http://maplab.cat/truke.

**Electronic supplementary material:**

The online version of this article (doi:10.1186/s12864-017-3631-8) contains supplementary material, which is available to authorized users.

## Background

The use of Excel in bioinformatics can lead to gene names converted to dates as the popular spreadsheet software auto-replaces gene symbols such as NOV1 by 1-nov or even 11/01/2016. Even though this issue was reported more than a decade ago and a shell script was released to check for data sanity [1], a recent paper by Ziemann et al. [2] pointed out the surprisingly high prevalence (about 20%) of corrupted gene symbols in Additional file 1 contained in genomics papers published in leading journals [2, 3].

The incredible persistence of this well-known bug contrasts with the lack of countermeasures; indeed, recovering the original gene names has been described as non feasible, thus irreversibly condemning corrupted data [1]. To try to overcome this issue we have developed Truke, an user friendly web tool to check for data integrity and, furthermore, to rollback tangled gene names to their original state.

## Implementation

**Fig. 1:**
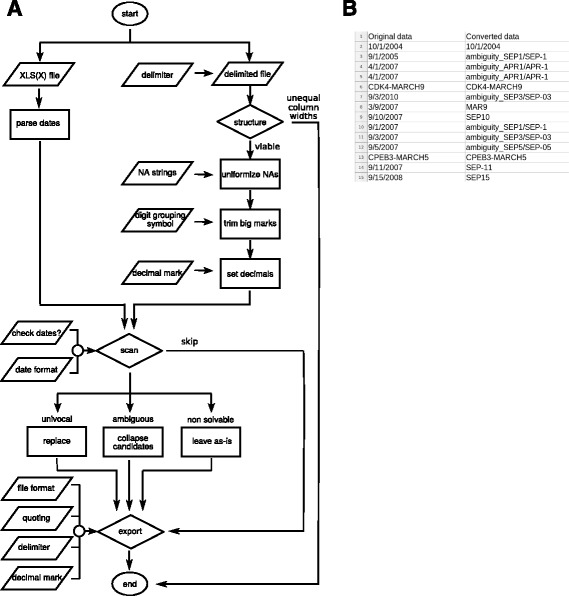
Data flow and usage example. **a**, Truke data flow. **b**, tabular data with the corrupted (*left*) and fixed (*right*) gene symbols. Data corresponds to the Ziemann’s [2] meta-analysis (Additional file 1) and was processed as if formatted by mm/dd/yyyy. Rows 1,6 and 13 exemplify dates which are not recoverable. Rows 3-5,7 and 10-12 depict dates which map to different gene symbols and therefore require further manual parsing. Rows 8, 9, 14 and 15 are unambiguous fixes

Truke is a Web tool to detect and fix Excel misconversions from plain text structured and XLS and XLSX files (Fig 1 a). To do so, Truke uses a previously built dictionary of gene symbols susceptible of being transformed to dates.

To generate the dictionary, we daily download all the approved gene symbols and synonyms from any species from the National Center for Biotechnology Information (NCBI) (ftp://ftp.ncbi.nlm.nih.gov/gene/DATA/) and run a regular expression to detect those resembling dates. Next, we reverse engineer the dates which might be alienized by Excel into these target gene symbols and add them to the dictionary. The default outcome is species-oblivious; human only and non-human gene symbol dictionaries may be specified.

We note that the correspondence between corruptible dates to gene symbols is not always one-to-one (e.g. it is not a bijection but a surjection). For instance, 09/01/2005 in a mm/dd/yyyy format can correspond to either SEP1 or SEP-1. In such cases, a warning is raised and the conflicting value will be tagged as ambiguity followed by every possible mapping.

Truke recognizes and checks for syllabic Excel-like dates, such as sep-8, and hyphen- and slash-separated dates, including dd[-/]mm[-/]yyyy, mm[-/]dd[-/]yyyy, yyyy[-/]mm[-/]dd and yyyy[-/]dd[-/]mm. Whilst selecting a single format is recommended (thus assuming consistency across the spreadsheet), Truke also offers an heuristic approach to deal with mixed data without specifying the date pattern.

Independently, Truke scans the data columnwise to replace regional setting-specific characters, such as the decimal symbol (comma or dot) and the digit grouping mark (i.e. the thousands separator, e.g. comma, dot or space, as in 10,000, 10.000 or 10 000). To do so, it employs a hierarchy of pattern matching and replacements: first, setting of the column delimiting field (i.e. comma in comma-separated values); second, digit grouping marks stripping; third, decimal elements replacement. The tool is sensitive to missing values.

Truke was built with R/shiny using an HTML and bootstrap2 front-end and deployed in a GNU/Linux server. Truke requires no installation and can be accessed with any web browser and operating system, including mobile devices and commodity computers.

## Results and discussion

To exemplify the use of Truke we have analyzed the plain-text version (Additional file 1) of the supplementary material of Ziemann *et al* [2] (Fig. 1 b). Once the queried file has been uploaded, Truke provides a preview of the top ten rows and the user may select among different formatting options including the date format (i.e. mm/dd/yyyy). If potentially conflicting data are detected, a warning advice will be generated and the dates will be renamed to gene symbols according to the selected date format. Dates univocally matching gene symbols, such as 9/3/2010 to MAR9, are transformed on the fly. Mappings with multiple counterparts, such as 09/01/2005 to either SEP1 or SEP-1, are tagged as ambiguous so they will require manual curation (e.g. selecting the appropriate gene symbol according to the species the data comes from). It should be also noted that Excel generated errors also depend on the computer’s regional and language settings. Truke may not be able to handle all the misidentifications, especially when mixed formats coexist. Although this situation should be very uncommon, the meta-analysis nature of the Ziemann et al. dataset is one of such cases and selecting either the dd/mm/yyyy or the mm/dd/yyyy date format will produce different results.

Unfortunately, this is not the only type of data corruption that Excel and other spreadsheet software may generate when importing structured data. Plain text files containing tabular data (text, dates, numbers, etc.) are non standard and may be differently read by Excel depending on regional or language settings of the user’s computer. Namely, field delimiters can be set to tabs, commas, semicolons or spaces, while the character specifying the decimal symbol varies depending on the location (with about half of the world using a dot and the other half a comma). Numbers can also be printed with thousands separators (comma, dot or space) for the sake of readability. Even numbers written using the so-called ‘scientific notation’ will show language dependent differences. This versatility can result in potentially conflicting combinations, seriously compromising data integrity. Truke can handle all these format variations in both the input and output files by using simple radio buttons and checkboxes.

## Conclusions

In summary, Truke provides a user friendly interface that allows the detection and correction of misidentified gene symbols, as well as on the fly file format conversion of structured data text files. Truke may be freely used without registration at http://maplab.cat/truke.

## Availability and requirements

Project name: TrukeProject home page: https://bitbucket.org/imallona/truke and http://maplab.cat/trukeOperating system(s): Platform independentProgramming language: R/shinyOther requirements: Modern web browserLicence: GNU General Public License (GPL)

## Additional file

Additional file 1
**Ziemann’s supplementary file.** Tab-separated, plain text version of the Ziemann et al. [2] supplementary file. (TSV 148 kb)

## Abbreviations

NCBI: National Center for Biotechnology Information
